# Evaluating the *Toxocara cati* extract as a therapeutic agent for allergic airway inflammation

**DOI:** 10.1002/iid3.1307

**Published:** 2024-06-11

**Authors:** Amin Bakhshani, Sima Parande Shirvan, Soheil Sadr, Mohsen Maleki, Alireza Haghparast, Hassan Borji

**Affiliations:** ^1^ Department of Pathobiology, Faculty of Veterinary Medicine Ferdowsi University of Mashhad Mashhad Iran

**Keywords:** allergic asthma, hygiene hypothesis, ovalbumin‐induced allergic airway inflammation, *Toxocara cati* somatic extract

## Abstract

**Background:**

The hygiene hypothesis suggests that early life exposure to helminth infections can reduce hypersensitivity in the immune system.

**Objective:**

The present study aims to evaluate the effects of *Toxocara cati* (*T. cati*) somatic products on allergic airway inflammation.

**Methods:**

Between 2018 and 2020, *T. cati* adult worms were collected from stray cats in Mashhad, Iran (31 out of 186 cats), and their somatic extract was collected. Thirty BALB/c mice were equally divided into three groups, including the OVA group (sensitized and challenged with ovalbumin), the somatic administered group (received somatic extract along with ovalbumin sensitization), and the PBS group (sensitized and challenged with phosphate buffer saline). Bronchoalveolar lavage (BAL) fluid was collected to assess the number of cells, and lung homogenates were prepared for cytokine analysis. Histopathological analysis of the lungs was performed, and inflammatory cells and mucus were detected. Cytokine levels (IL‐4, IL‐5, IL‐10) were measured using enzyme‐linked immunosorbent assay (ELISA), and ovalbumin‐specific immunoglobulin E (IgE) levels were determined using a capture ELISA.

**Results:**

The somatic group significantly decreased regarding the lung pathological changes, including peribronchiolitis, perivasculitis, and eosinophil influx, compared to the group treated with ovalbumin alone. These changes were accompanied by a decrease in proinflammatory cytokines IL‐4 and IL‐5 and an increase in the anti‐inflammatory cytokine IL‐10, indicating a shift toward a more balanced immune response. The number of inflammatory cells in the BAL fluid was also significantly reduced in the somatic group, indicating a decrease in inflammation.

**Conclusion:**

These preclinical findings suggest that in experimental models, *T. cati* somatic extract exhibits promising potential as a therapeutic agent for mitigating allergic airway inflammation. Its observed effects on immune response modulation and reduction of inflammatory cell infiltration warrant further investigation in clinical studies to assess its efficacy and safety in human patients.

## Highlights

- Allergic asthma is linked to Th2 cell responses, while corticosteroid side effects discourage patients.
- The hygiene hypothesis suggests helminth infections could reduce immune hypersensitivity.
- The administration of *Toxocara cati* somatic extract significantly decreased lung pathological changes associated with allergic airway inflammation, including peribronchiolitis, perivasculitis, and eosinophil influx.
- This reduction was accompanied by a shift in cytokine levels, with decreases in proinflammatory cytokines IL‐4 and IL‐5 and an increase in the anti‐inflammatory cytokine IL‐10, suggesting a rebalancing of the immune response.
- The somatic extract treatment significantly reduced inflammatory cell infiltration in the bronchoalveolar lavage fluid, indicating a decrease in overall inflammation within the airways.

## Graphical abstract

The hygiene hypothesis suggests that helminth infections can reduce hypersensitivity in the immune system. This study aimed to evaluate the effects of *Toxocara cati* somatic products on allergic airway inflammation. These findings suggest that *T. cati* somatic extract has the potential as a therapeutic agent for attenuating allergic airway inflammation by modulating immune responses and reducing inflammatory cell infiltration.

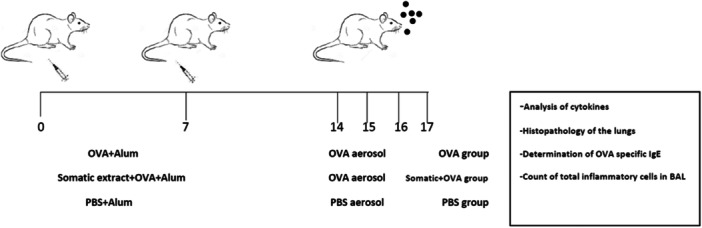

## 1 INTRODUCTION

Many diseases are caused by parasitic infections.^1^ A common intestinal nematode found in cats, *Toxocara cati*, is associated with asthma in humans.^2^ *Toxocara cati* goes through several stages in its lifecycle.^3^ Adult worms stay inside the intestines of infected cats by excreting eggs into the environment through the cat's feces.^4^ It takes only a few weeks for these eggs to become infective after being embryonated in the environment.^5^ In somatic migration, cats ingest the infective eggs, which release larvae into the intestine that invade the abdominal wall and travel through various organs, including the liver and lungs.^6^ Coughing up and swallowing the larvae and reaching the lungs completes the life cycle.^7^ If the environment is contaminated with *T. cati* eggs, humans may be inadvertently exposed to *T. cati* eggs.^8^ After that, larvae will be triggered to migrate into the human body when they are ingested by humans.^9^ Larvae in humans cannot reach maturity compared to feline hosts.^10^ On the other hand, somatic migration is the process by which larvae move through various organs within the body, including the liver and blood vessels.^11^

Humans may experience various clinical manifestations due to somatic migration, commonly known as visceral larva migrants.^12^ During their migration through tissues, the larvae trigger immune reactions that lead to inflammations.^13^ Some symptoms may be experienced by those affected, including fevers, fatigue, and discomfort.^14^ Symptoms of severe infestations may include hepatomegaly and vision impairment caused by larvae migrating through the eyes.^15^, ^16^

In allergic asthma, an inflammatory disorder of the airways, immune dysregulation leads to exaggerated responses to otherwise harmless environmental allergens, resulting in recurrent wheezing, breathlessness, chest tightness, and coughing.^17^ Key immune cells involved in this process include T helper 2 (Th2) cells, which release cytokines such as interleukin‐4 (IL‐4) and IL‐5, promoting the production of IgE antibodies and the recruitment of eosinophils.^18^, ^19^ Additionally, regulatory T cells (Tregs) play a crucial role in maintaining immune tolerance and controlling excessive inflammation by releasing anti‐inflammatory cytokines like IL‐10.^20^ These cellular signaling pathways orchestrate the inflammatory cascade underlying allergic lung inflammation, contributing to the pathogenesis of allergic asthma.^21^, ^22^

Research on the potential link between intestinal nematodes and asthma has increased over the past few years due to the intriguing interplay between parasitic infections and human health.^23^, ^24^ There has been some evidence of a link between *T. cati* infection and asthma, especially during the larval migration.^25^ During the larvae's migration, immune responses could be triggered within the human body, affecting asthma development.^26^, ^27^ When the immune system responds to migrating larvae by activating an inflammatory response, it may inadvertently cause chronic inflammation and remodeling of the airways, typical asthma symptoms, in an attempt to eliminate the parasites.^28^, ^29^ Despite all, *T. cati* may be associated with asthma in its own right, and it is now the focus of researchers to understand the impact of specific antigens of *T. cati* on asthma development.^30^, ^31^, ^32^

Finding antigens that drive proinflammatory responses may make targeted therapies to modulate immune reactions and mitigate asthma symptoms possible. A new asthma therapeutic strategy could be developed if antigens with anti‐inflammatory properties are identified. The present study aims to evaluate the effects of *T. cati* somatic products on allergic airway inflammation.

## 2 METHODS AND MATERIALS

### 2.1 Collecting of *T. cati* worms and preparation of their somatic extract

With authorities' permission from the Iranian Environmental Health Organizations, 186 stray cats from different districts of Mashhad, were collected using baited cage traps. After collection, they were moved to the veterinary hospital of the Faculty of Veterinary Medicine of Ferdowsi University of Mashhad and kept in individual cages with unique identification numbers. Then, the formalin‐ether sedimentation method (using fecal samples) was employed to identify *T. cati* infection. A total of 31 cats were infected with *T. cati* (21% infection in Mashhad). They were euthanized intravenously by sodium thiopental (12.5 mg/kg) and potassium chloride (1–2 mmol/kg) and autopsied within an hour of the mentioned process. Adult worms of *T. cati* were separated and put in PBS. *Toxocara cati* adult worms were recognized using the method described by Yamaguti et al. in 1961.^33^ The worms were washed three times and homogenized using a sterile PBS containing antibiotics (penicillin/streptomycin). The worm homogenate was centrifuged at 10,000×*g*, 4°C for 10 min, and the supernatants were collected into new sterile tubes.

### 2.2 Remove the lipopolysaccharide from the prepared somatic extract

Triton X‐114‐assisted LPS‐extraction method was used to remove LPS. TX‐114 was added to the somatic extract to a final TX‐114 concentration of 2% v/v. The solution was incubated at 4°C for 30 min with constant stirring. Subsequently, the sample was transferred to a water bath set at 37°C and incubated for 10 min, followed by centrifugation at 20,000*g* for 20 min at 37°C. Pipetting separated the upper part containing the protein from the TX‐114 layer. To increase LPS removal efficiency, the extraction procedure was repeated three times. Bradford assay (Bio‐Rad) determined the total protein concentration. The LPS‐free somatic extract was aliquoted and stored at −80°C until.^34^

### 2.3 Experimental design and grouping

Thirty 5‐week‐old specified pathogen‐free female BALB/c mice with a range weight of 16−20 g were obtained from Razi Vaccine and Serum Research Institute and maintained in animal housing facilities At the Ferdowsi University of Mashhad throughout the experiment. This study included three groups of mice consisting of 10 animals per group.

**Figure 1:**
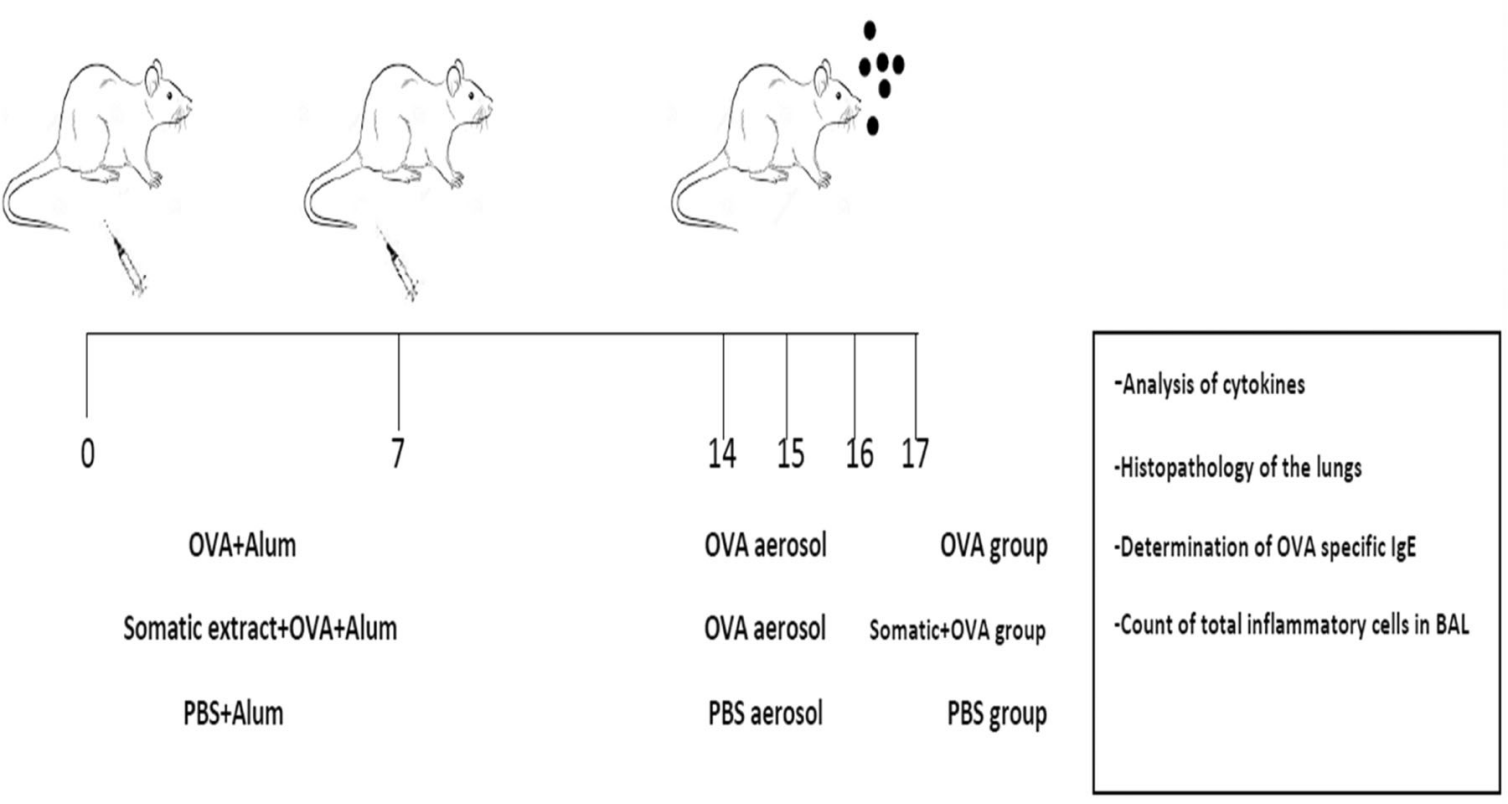
Experimental protocol for induction of allergic airway inflammation and treatment scheme. Mice were divided into three groups: OVA‐treated, OVA + somatic, and PBS. The OVA‐treated group received OVA sensitization followed by nebulized OVA challenges, while the OVA + somatic group received OVA sensitization with somatic extract and the same nebulized challenges. The PBS group received PBS sensitization and nebulized PBS challenges. All groups were euthanized on Day 17 for analysis.

The first group was the OVA‐treated group, and mice were sensitized intraperitoneally (IP) with two injections of OVA emulsified in AL(OH)3 on Days 0 and 7. Seven days after the last sensitization, a challenge was performed by exposing the mice for 30 min with 1% OVA in PBS generated by a nebulizer at Days 14, 15, and 16. The second group was the OVA + somatic group, and mice received 20 μg somatic extract during sensitization with OVA‐alum on Days 0 and 7. Seven days after the last sensitization, a challenge was performed by exposing the mice for 30 min with 1% OVA in PBS generated by a nebulizer at Days 14, 15, and 16. Finally, the third group was the PBS group, and mice were sensitized with 0.5 mL PBS‐alum on Days 0 and 7 and challenged by nebulizer inhalation of PBS on Days 14, 15, and 16. Mice of all groups were euthanized on Day 17. The experimental design is schematically represented in Figure 1 (Figure 1).

### 2.4 Collection of bronchoalveolar lavage fluid

Bronchoalveolar Lavage (BAL) fluid was collected to assess the number of cells. In the first step, a cannula was put in their trachea, and PBS (3 × 0.4 mL) was injected into their lungs. After that, 1 mL of BAL fluid was extracted using the negative pressure and centrifuged at 400*g* for 10 min at 4°C. Then, in 100 μL of PBS, another round of suspension was done for cell pallets, and for further analysis, cell‐less supernatants were kept at −20°C. Ultimately, the number of inflammatory cells in the BAL was quantified using a hemocytometer.

### 2.5 Preparing lung homogenate

After collecting the BAL, the lungs were harvested and organized in two sets. The first lung set was used for histopathological analysis (fixed in 10% phosphate‐buffered formalin). The second set was employed to detect cytokine. Thus, they were frozen in liquid nitrogen and kept at −80°C. Before the cytokine detection process, the frozen lungs were thawed and weighed. After that, 100 pg/100 mg of lung tissue homogenization buffer containing Triton X‐100, Tris‐CL (1 M, pH 9), and KCL (0.5 M). Next, the homogenates were centrifuged at 10,000×*g* at 4°C for 10 min. Finally, an Enzyme‐Linked Immunosorbent Assay (ELISA) was conducted to analyze the cytokine of the supernatants.

### 2.6 Histopathology of the lungs

To assess the inflammatory cell infiltration, lung tissues were set in paraffin (after 24 h of formalin fixation). Then, they were cut into 5 μm sections and stained with hematoxylin−eosin (H&E).

Histological changes (peribronchiolitis, perivascular infiltrate, goblet cell metaplasia in bronchioles, and eosinophil influx) were semiquantitatively and blindly scored from absent (0), minimal (1), slight (2), moderate (3), marked (4) to severe (5).

### 2.7 Cytokines detection

IL‐4, IL‐5, and IL‐10 levels were measured in BAL fluid and lung tissue homogenates using a sandwich ELISA (BT Lab kits) according to the manufacturer's recommendations (Bioassay Technology Laboratory). Detection limits for IL‐10, IL‐5, and IL‐4 were 2.48, 0.51, and 2.53 pg/mL, respectively.

### 2.8 Ovalbumin‐specific immunoglobulin E (IgE)

Determination of OVA‐specific IgE levels was performed using a capture ELISA (Bioassay Technology Laboratory). The assay's detection limit was 2.56 ng/mL.

### 2.9 Statistical analysis

Prism 6.01 (Graghpad) was used for data analysis. Using Kruskal−Wallis and one‐way ANOVA, the statistical significance between groups was calculated, considering *p*‐values less than 0.05 as significant.

## 3 RESULTS

### 3.1 Histopathology of the lungs

Table 1 shows the pathological changes observed in the lungs of BALB/c mice in three studied groups. The presence of *T. cati* somatic products during OVA sensitization substantially decreased pathological lesions in a somatic administered group compared with the OVA group. Peribronchiolitis, perivasculitis, and eosinophil influx were significantly decreased in mice from the somatic + OVA group compared with the OVA group. Goblet cell metaplasia in bronchioles was not significantly different between somatic + OVA and OVA groups (Table 1).

**Table 1:** Pathological changes in the lungs of BALB/c mice in the study groups.

Pathological changes	Somatic + OVA	OVA	PBS
Peribronchiolitis	1.6 ± 0.74*	2.3 ± 0.5	0
Perivasculitis	1.7 ± 1.03*	2.8 ± 0.9	0
Goblet cell metaplasia	2 ± 0.7	2.7 ± 1.2	0
Eosinophils influx	1.75 ± 0.7*	3.2 ± 0.4	0

*Note*: Peribronchiolitis, perivasculitis, and eosinophils influx were significantly (*) decreased in mice from the somatic + OVA group compared with mice from the OVA group. However, goblet cell metaplasia in bronchioles was not significantly different between somatic + OVA and OVA groups. The histopathological scores were: absent (0), minimal (1), slight (2), moderate (3), marked (4), severe (5).

Mice treated with OVA to induce allergic airway inflammation (OVA group) showed a slight to moderate peribronchiolar and perivascular infiltrate consisting of mononuclear cells and eosinophils. Goblet cell metaplasia in bronchioles was moderate.

**Figure 2:**
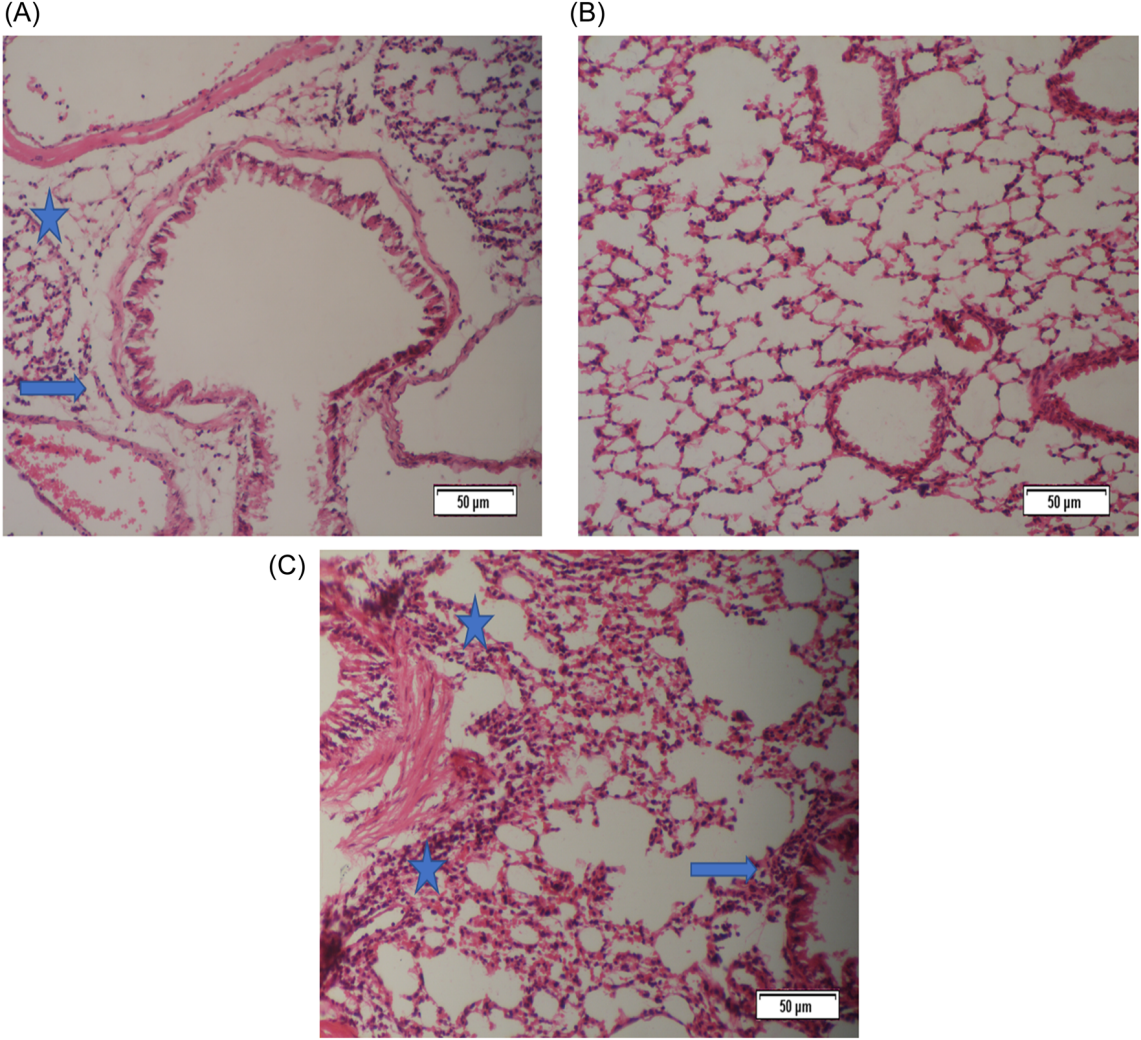
Peribronchiolitis, perivasculitis, and eosinophil influx were significantly decreased in mice from the somatic + OVA group compared with the OVA group. Mice treated with OVA to induce allergic airway inflammation (OVA group) showed a slight to moderate peribronchiolar and perivascular infiltrate consisting of mononuclear cells and eosinophils. The lungs of mice from the somatic + OVA group showed minimal to slight peribronchiolitis, perivasculitis, and eosinophil influx, while the goblet cell metaplasia in bronchioles was slight to moderate. (A) Histopathological slides of somatic + OVA group stained with H&E (×20). (B) Histopathological slides of the control group stained with H&E (×20). (C) Histopathological slides of the OVA group were stained with H&E (×20). Blue arrows: Peribronchiolitis, blue stars: Perivasculitis. H&E, hematoxylin−eosin.

**Figure 3:**
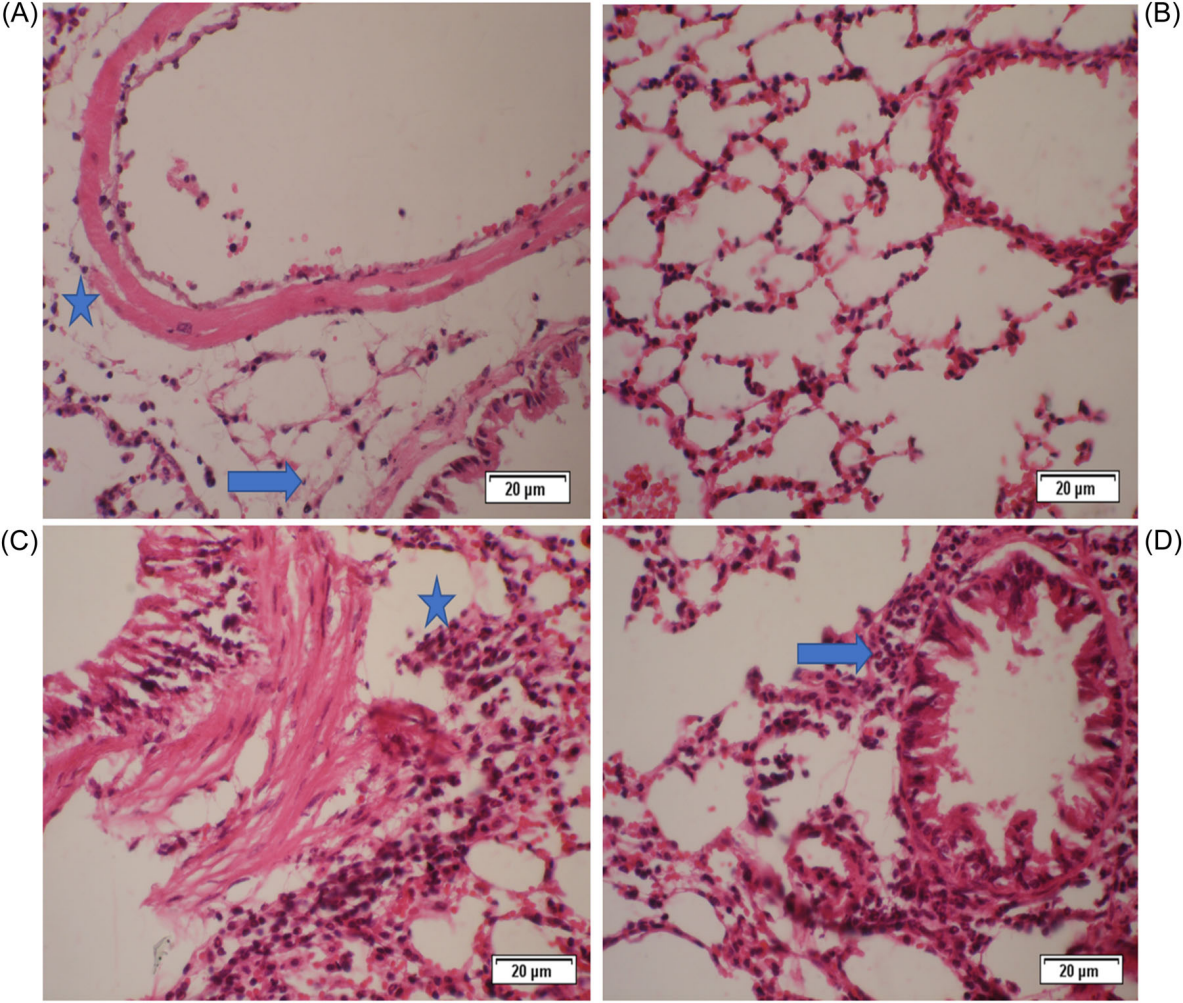
Peribronchiolitis, perivasculitis, and eosinophil influx were significantly decreased in mice from the somatic + OVA group compared with the OVA group. Mice treated with OVA to induce allergic airway inflammation (OVA group) showed a slight to moderate peribronchiolar and perivascular infiltrate consisting of mononuclear cells and eosinophils. The lungs of mice from the somatic + OVA group showed minimal to slight peribronchiolitis, perivasculitis, and eosinophil influx, while the goblet cell metaplasia in bronchioles was slight to moderate. (A) Histopathological slides of somatic + OVA group stained with H&E (×40). (B) Histopathological slides of the control group stained with H&E (×40). (C, D) Histopathological slides of the OVA group were stained with H&E (×40). Blue arrows: Peribronchiolitis, blue stars: Perivasculitis. H&E, hematoxylin−eosin.

**Figure 4:**
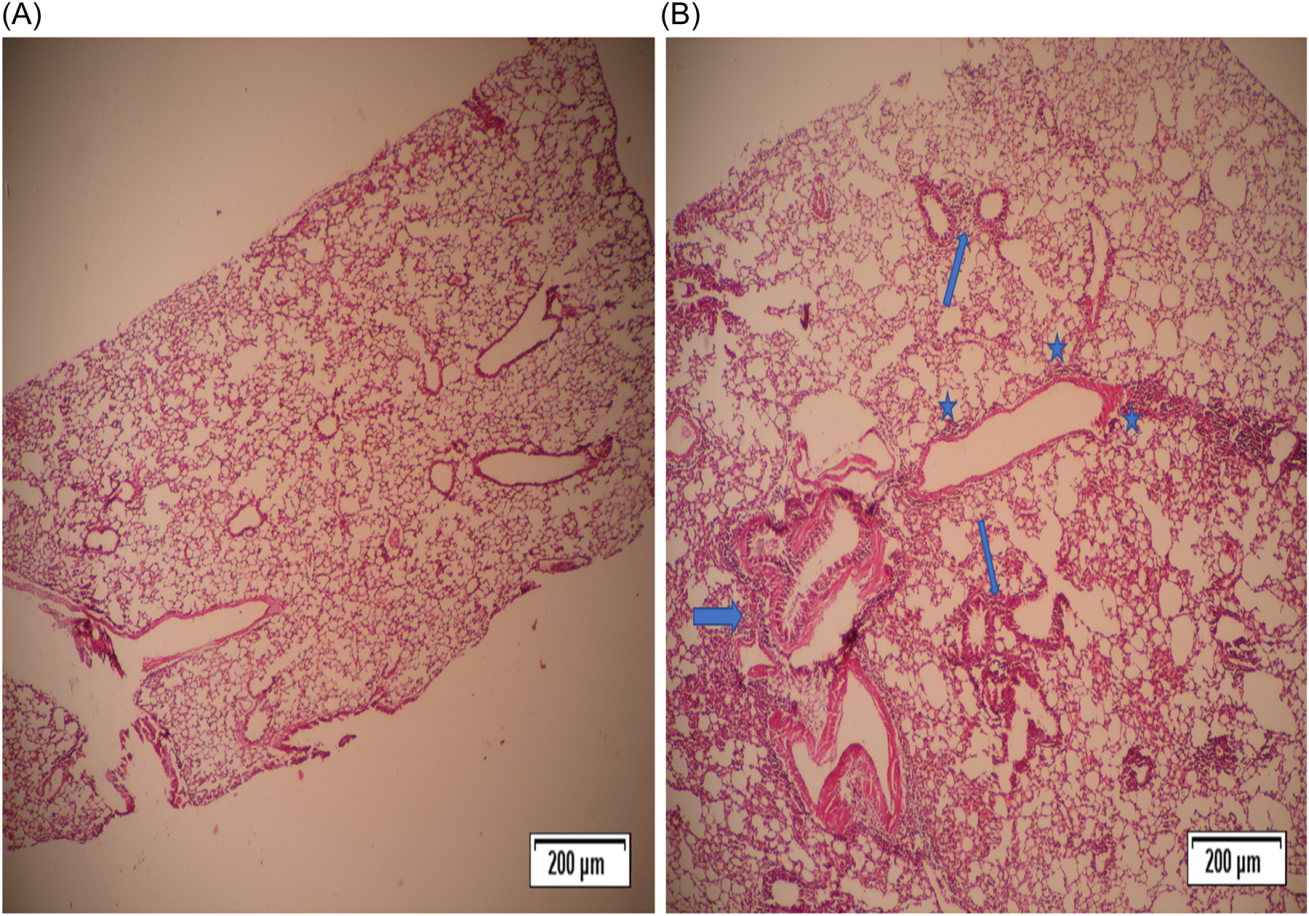
Peribronchiolitis, perivasculitis, and eosinophil influx were significantly decreased in mice from the somatic + OVA group compared with the OVA group. Mice treated with OVA to induce allergic airway inflammation (OVA group) showed a slight to moderate peribronchiolar and perivascular infiltrate consisting of mononuclear cells and eosinophils. The lungs of mice from the somatic + OVA group showed minimal to slight peribronchiolitis, perivasculitis, and eosinophil influx, while the goblet cell metaplasia in bronchioles was slight to moderate. (A) Histopathological slides of the control group stained with H&E (×4). (B) Histopathological slides of the OVA group were stained with H&E (×4). Blue arrows: Peribronchiolitis, blue stars: Perivasculitis. H&E, hematoxylin−eosin.

**Figure 5:**
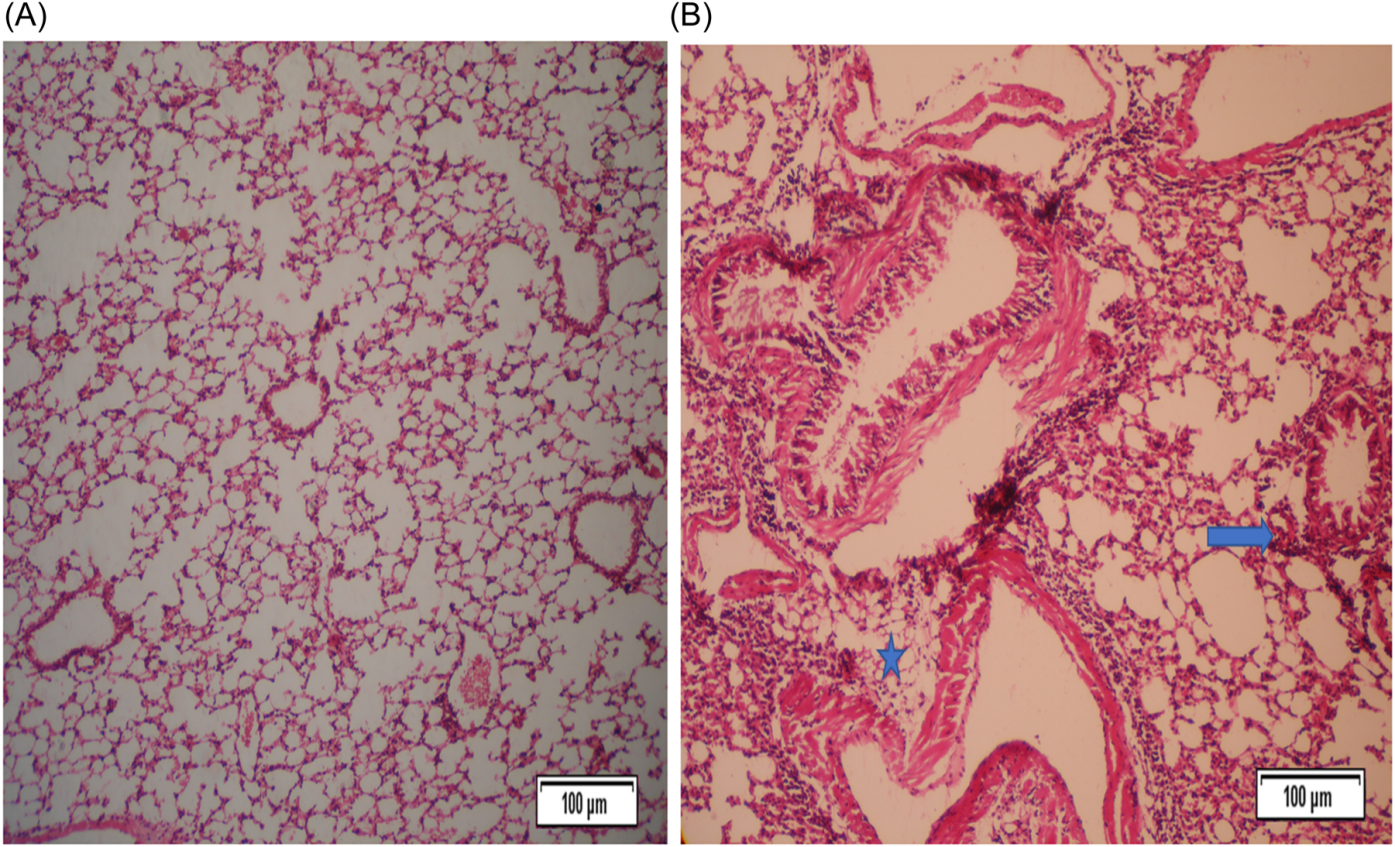
Peribronchiolitis, perivasculitis, and eosinophil influx were significantly decreased in mice from the somatic + OVA group compared with the OVA group. Mice treated with OVA to induce allergic airway inflammation (OVA group) showed a slight to moderate peribronchiolar and perivascular infiltrate consisting of mononuclear cells and eosinophils. The lungs of mice from the somatic + OVA group showed minimal to slight peribronchiolitis, perivasculitis, and eosinophil influx, while the goblet cell metaplasia in bronchioles was slight to moderate. (A) Histopathological slides of the control group stained with H&E (×10). (B) Histopathological slides of the OVA group were stained with H&E (×10). Blue arrows: Peribronchiolitis, blue stars: Perivasculitis. H&E, hematoxylin−eosin.

The lungs of mice from the somatic + OVA group showed minimal to slight peribronchiolitis, perivasculitis, and eosinophil influx, while the goblet cell metaplasia in bronchioles was slight to moderate. In the PBS group, no histological lesions were observed (Figures 2, 3, 4, 5).

### 3.2 Cytokines in BAL and lung homogenate

IL‐4 and IL‐5 levels were significantly increased in OVA‐sensitized mice compared to the PBS group. Results indicate a significant decrease in IL‐4 and IL‐5 levels In BAL and lung homogenate of mice from the somatic + OVA group compared with OVA group mice. The results showed that the administration of somatic extract of *T. cati* suppressed Th2 cytokines production.

**Figure 6:**
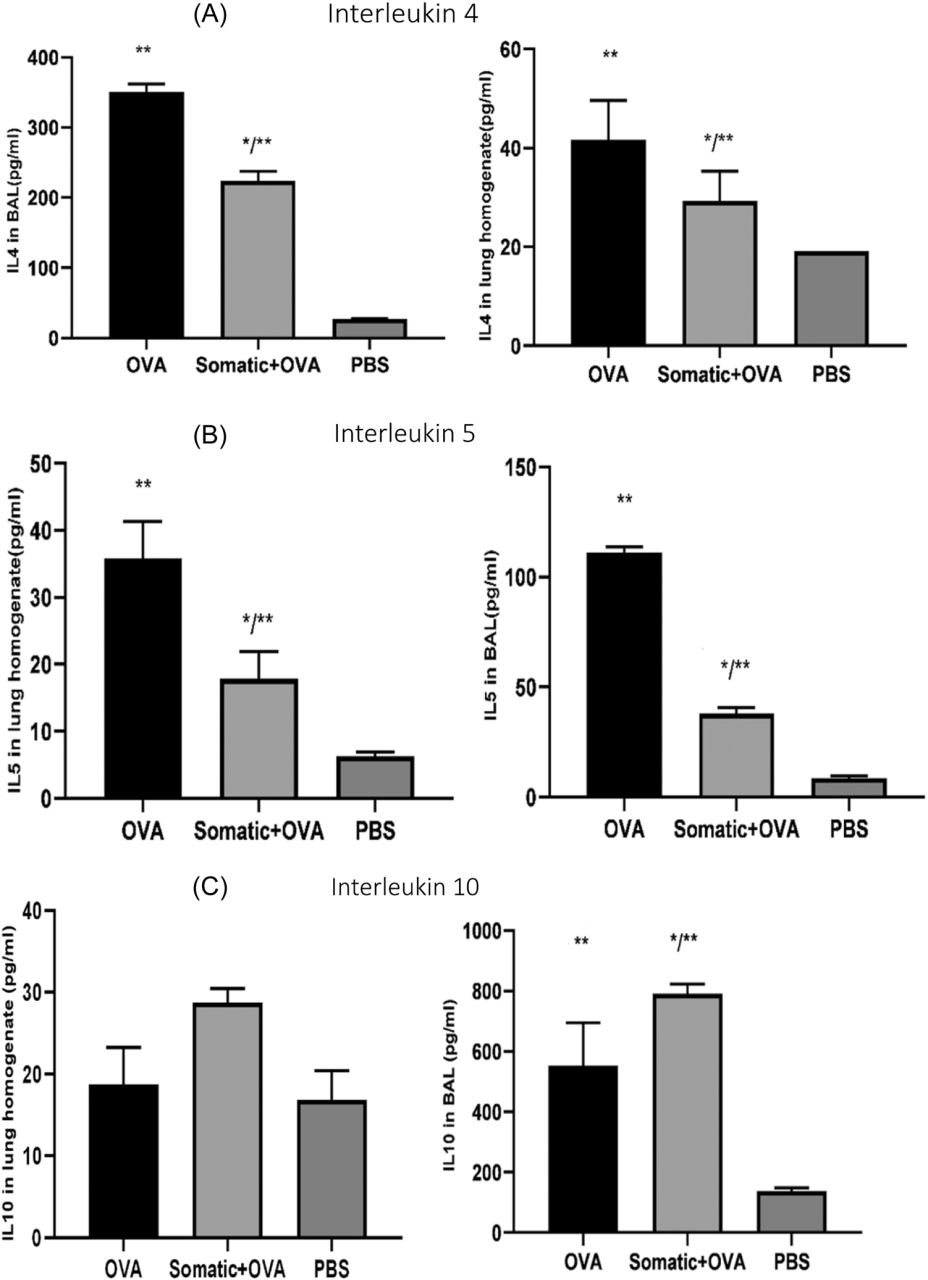
Cytokine levels IL‐4 (A), IL‐5 (B), and IL‐10 (C) in BAL and lung homogenate were measured in 30 5‐week‐old specified pathogen‐free female BALB/c mice, including three groups of mice consisting of 10 animals per group. The first group was the OVA‐treated group, and mice were sensitized intraperitoneally (IP) with two injections of OVA emulsified in AL(OH)3 on Days 0 and 7. The second group was the OVA + somatic group, and mice received 20 μg somatic extract during sensitization with OVA‐alum on Days 0 and 7. Finally, the third group was the PBS group, and mice were sensitized with 0.5 mL PBS‐alum on Days 0 and 7 and challenged by nebulizer inhalation of PBS on Days 14, 15, and 16. Somatic extract of *Toxocara cati* significantly suppressed Th2 cytokines via upregulation of IL‐10. One star (*) means of existing a significantly different (*p* < .05) from group OVA. Kruskal−Wallis and one‐way ANOVA tests were used. Two stars (**) means of existing a significantly different (*p* < .05) from group PBS. Kruskal−Wallis and one‐way ANOVA tests were used. BAL, bronchoalveolar lavage.

The level of IL‐10 In BAL of mice from the somatic + OVA group significantly increased compared with mice from OVA and PBS groups. The suppressive effects of *T. cati* somatic extract on OVA‐induced airway inflammation were significantly associated with the upregulation of IL‐10 (Figure 6).

### 3.3 Ovalbumin‐specific IgE in BAL and lung homogenate

**Figure 7:**
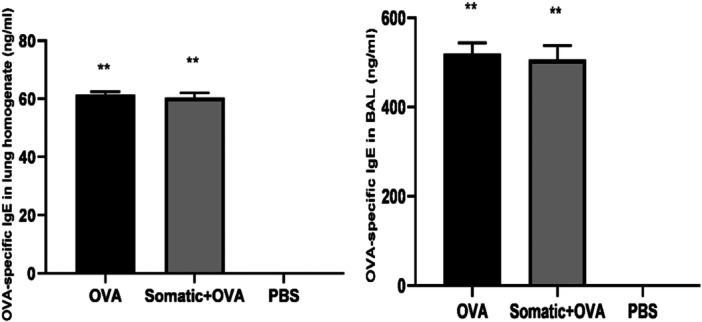
OVA‐specific IgE concentration in lung homogenate and bronchoalveolar lavage (BAL) of BALB/c mice in study groups. Two stars (**) means of existing a significantly different (*p* < .05) from group PBS. Kruskal−Wallis and one‐way ANOVA tests were used.

Figure 7 shows the levels of OVA‐specific IgE in mice's lung homogenate and BAL from the study groups. The OVA group showed higher OVA‐specific IgE levels than the somatic + OVA group, but this difference was insignificant (Figure 7).

### 3.4 Total inflammatory cells, including eosinophils in BAL

**Figure 8:**
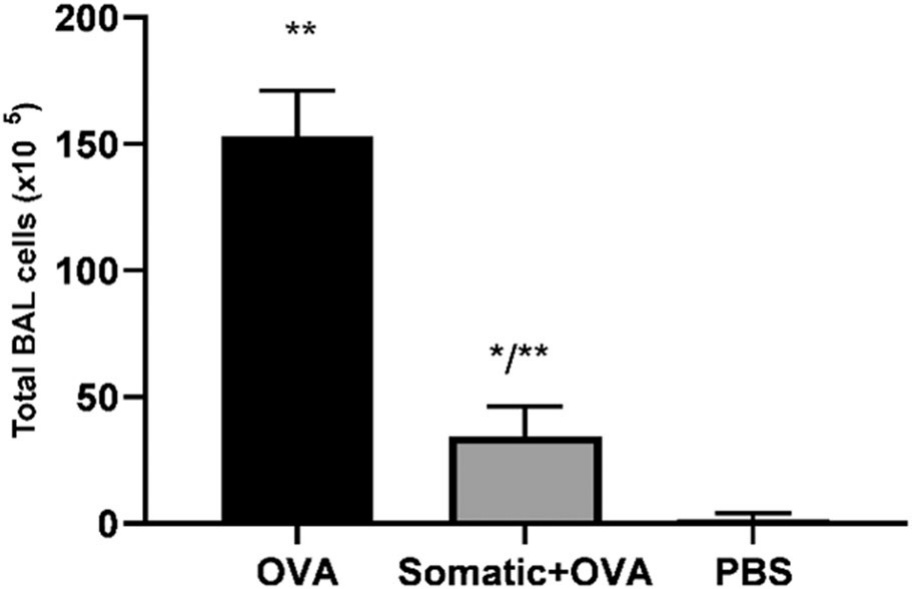
BAL cells were analyzed in three studied groups. One star (*) means of existing a significantly different (*p* < .05) from group OVA. Kruskal−Wallis and one‐way ANOVA tests were used. Two stars (**) means of existing a significantly different (*p* < .05) from group PBS. Kruskal−Wallis and one‐way ANOVA tests were used. BAL, bronchoalveolar lavage.

The number of inflammatory cells in BAL from mice that received a somatic extract, OVA, and PBS groups is shown in Figure 8. Results indicate that receiving a somatic extract resulted in a significantly decreased number of inflammatory cells in BAL compared to the OVA group.

The total inflammatory cells were decreased in the BAL fluid of mice that received 20 μg somatic extract of *T. cati* during sensitization along with OVA + alum. The total cell count in the BAL of mice from study groups showed the same results in pathological sections.

The highest number of cells in BAL fluid was obtained from the OVA group due to the increasing number of eosinophils. In contrast, *T. cati* somatic extract decreases allergic responses by reducing the infiltration of inflammatory cells into BAL fluid (Figure 8).

## 4 DISCUSSION

The present study was designed to examine whether *T. cati* somatic extract can potentially ameliorate allergic airway inflammation in an attempt to treat asthma using a murine model of asthma. Despite the complexity of its etiology, allergic asthma remains one of the most challenging health problems around the world for several reasons, including the lack of efficacy of current treatments and the adverse effects of long‐term corticosteroid use.^35^, ^36^, ^37^ In the last few years, helminth infections have received increasing attention for their potential immunomodulatory effects, and researchers have explored alternative therapeutic approaches in pursuit of those purposes.^38^, ^39^ As outlined in the hygiene hypothesis, exposure to certain pathogens like helminths early in life may benefit the immune system, reducing hypersensitivity in subsequent stages of life, therefore providing a possibility to develop new therapeutic approaches.^40^, ^41^, ^42^ An important objective of this study was to elucidate the mechanisms underlying the immunomodulatory properties of *T. cati* somatic extract to alleviate allergic airway inflammation.

The current study provided promising results, which support the hypothesis that *T. cati* somatic extract may have therapeutic potential as a therapeutic adjunct for the treatment of allergic asthma, as indicated by the results of this study. There was a significant reduction in pathological lung changes when the *T. cati* somatic extract was administered during sensitization compared with the ovalbumin group. The somatic extract reduced peribronchiolitis, perivasculitis, and eosinophil influx in the somatic group, suggesting that the extract had an additional anti‐inflammatory effect. This is in agreement with the occurrence of an attenuated inflammatory response.

Asthma is a highly symptomatic disease attributed to the imbalance of cytokines in the body, which can play a critical role in orchestrating the immune response. Using somatic extract as a therapeutic option showed decreased levels of proinflammatory cytokines IL‐4 and IL‐5 associated with allergic asthma, including airway hyperactivity and mucus production, associated with exacerbated asthma symptoms. In the somatic extract group, the Th2 cytokines were downregulated, indicating that a less inflammatory state is being achieved in the immune response due to the downregulation. Moreover, IL‐10 levels increased, indicating a shift toward a more balanced immune response, while there was also an increase in the anti‐inflammatory cytokine IL‐2. As a consequence of the upregulation of IL‐10, which is known for its anti‐inflammatory properties and well‐known for its ability to regulate allergic inflammation, *T. cati* somatic extract may have further anti‐inflammatory effects.

In addition to the significant reduction in ovalbumin‐specific IgE levels after administration of the somatic extract, an immunoglobulin important to allergic sensitization and modulation of cytokine profiles was also significantly reduced. A decrease in IgE levels could indicate inhibition of the allergic response. The study's results indicate that ovalbumin‐specific IgE levels have decreased, which supports the theory that helminth infections suppress hypersensitivity reactions by inducing immune‐regulatory mechanisms.

The findings of the current study demonstrate a significant reduction in the levels of key inflammatory mediators, namely IL‐4 and IL‐5, in the BAL and lung homogenate of mice treated with *T. cati* somatic extract alongside ovalbumin sensitization, compared to OVA‐sensitized mice without treatment. This suppression of Th2 cytokine production suggests a mechanism by which *T. cati* somatic extract exerts its anti‐inflammatory effects, potentially by modulating the immune response toward allergens. Notably, the upregulation of IL‐10 levels in the BAL of treated mice further supports the immunomodulatory role of *T. cati* somatic extract, highlighting its ability to promote regulatory pathways and dampen excessive inflammation. Additionally, although not reaching statistical significance, a trend toward reduced OVA‐specific IgE levels in treated mice's lung homogenate and BAL underscores the potential of *T. cati* somatic extract in modulating allergen‐specific immune responses.

Further evidence indicates a reduction in inflammation in the BAL fluid, indicating that the somatic extracts have anti‐inflammatory properties, suggesting a reduction in pulmonary inflammation. According to the histopathological findings, inflammatory cell infiltrates decreased following treatment with somatic extracts. The findings of this study demonstrated that inflammatory cells, such as eosinophils, which are markers of allergic inflammation, may be reduced by somatic extracts, which modulate the immune response.

To make a reasonable conclusion based on the findings of this study, it is necessary to acknowledge that the study has several limitations. This study employed a mouse model of asthma to assess the therapeutic potential of *T. cati* somatic extracts. One limitation of the current study is the reliance on a single experiment repetition, albeit with a higher number of animals, which may limit the generalizability of the findings and the robustness of the conclusions. Furthermore, while efforts were made to mitigate endotoxin contamination, the lack of final confirmation for eliminating LPS introduces a potential source of variability that could impact the interpretation of the results. Future studies in humans will be required to confirm the efficacy of the extracts. Additionally, no specific components of the somatic extracts were identified as causing the observed effects. Bioactive molecules present in the extract should be identified and characterized to make valuable progress toward understanding the extract's underlying mechanisms of action. One limitation of the current study is the lack of comprehensive assessment of Th1 immune responses, including their respective cytokines, such as TNF‐alpha and interferon‐gamma, due to resource constraints. However, it is noteworthy that the current study offers a complementary perspective that enhances the understanding of the underlying mechanisms involved in allergic Th2‐high phenotypic asthma alongside histopathological examinations, which provided valuable insights.

Further studies should investigate the mechanisms explaining the lack of significant differences in IgE levels despite significant differences in cellular infiltration and Th2 cytokine production. Various mechanisms could be involved, such as the nematode extract's different mechanisms of action that may not directly influence IgE antibody production. The effects might involve pathways more closely associated with cellular inflammation and cytokine regulation than with IgE synthesis, possibly TGF‐B or specific MAPK component pathways. Future studies should conduct mechanistic investigations, such as western blot analysis in lung homogenates, to enhance the study's value. It is also important to note the sensitivity of the IgE assays employed in the study.

This finding aligns with the recognized characteristic of effective allergen‐specific immunotherapy in humans, wherein the presence of IL‐10‐producing Treg cells decreases allergic symptoms. The suggested process involves helminths‐inducing Tregs, which promote immune tolerance and reduce Th2 responses, suppressing inappropriate inflammatory reactions linked to allergic asthma.^43^, ^44^ Future studies could include measuring Foxp3 levels (a Tregs marker) in the homogenate by western blot analysis to elucidate this mechanism further. Anticipated results might reveal higher Foxp3 levels in the somatic group homogenate samples, providing valuable insights into the regulatory pathways associated with the increased IL‐10.

## 5 CONCLUSION

In conclusion, the findings from the present experimental trials provide compelling evidence for the potential therapeutic effects of *T. cati* somatic extract in allergic asthma. In these trials, the observed attenuation of allergic airway inflammation, modulation of cytokine profiles, inhibition of IgE production, and reduction in inflammatory cell infiltrates support the notion that helminth‐derived molecules may offer an alternative or adjunctive therapeutic approach for allergic asthma. Further research is warranted to elucidate the precise mechanisms of action and to evaluate the safety and efficacy of *T. cati* somatic extract in human subjects.

## AUTHOR CONTRIBUTIONS

**Hassan Borji and Mohsen Maleki**: Conceptualization; supervision. **All authors**: Methodology; formal analysis and investigation; Writing—original draft preparation; Writing—review and editing.

## CONFLICT OF INTEREST STATEMENT

The authors declare no conflict of interest.

## ETHICS STATEMENT

All applicable international, national, and/or institutional guidelines for the care and use of animals were followed. The study procedure has been approved by the ethical committee of the Animal Welfare Committee at the Ferdowsi University of Mashhad (ID: 40436).

## Data Availability

The data sets generated during and/or analyzed during the current study are available from the corresponding author on reasonable request.
